# Brain Metastases from Adult Sarcomas: A Retrospective Cohort Study from the Hellenic Group of Sarcomas and Rare Cancers (HGSRC)

**DOI:** 10.3390/jcm10245978

**Published:** 2021-12-20

**Authors:** Stefania Kokkali, Louiza Vini, Anastasia Stergioula, Anastasios Kyriazoglou, Nikolaos Vassos, Ioannis Boukovinas

**Affiliations:** 1 Department of Medical Oncology, Saint-Savvas Anticancer Hospital, 11522 Athens, Greece; stefaniakokkali8@gmail.com; 2 Department of Radiation Oncology, Athens Medical Center, 15125 Marousi, Greece; vinilouiza@gmail.com; 3 Department of Radiation Oncology, “Iaso” Hospital, 15123 Marousi, Greece; nastasia_gr@hotmail.com; 4 Department of Tomotherapy-Stereotactic Radiosurgery, “Iatropolis Medical Group”, 15231 Chalandri, Greece; 5 Medical Oncology Unit, Department of Clinical Therapeutics, General Hospital Alexandra, 11528 Athens, Greece; tassoskyr@gmail.com; 6 Division of Surgical Oncology and Thoracic Surgery, Mannheim University Medical Center, University of Heidelberg, 68167 Mannheim, Germany; 7 Department of Medical Oncology, Bioclinic Hospital, 54622 Thessaloniki, Greece; ibouk@bioncology.gr

**Keywords:** brain metastases, cerebral metastases, soft tissue sarcoma, bone sarcoma

## Abstract

Brain metastases are rare events in patients with sarcoma and the available information is relatively limited. We retrospectively reviewed medical records of patients with sarcoma who developed brain metastases between April 2010 and April 2020 in six centers. Thirty-four adult patients were included with a median age at brain metastases diagnosis of 55.5 years (range, 18–75). The primary sarcomas originated either from soft tissue (*n* = 27) or bone (*n* = 7) and the most common subtypes were leiomyosarcoma (*n* = 8), Ewing sarcoma/peripheral neuroectodermal tumor (PNET) (*n* = 7) and osteosarcoma (*n* = 3). Most primary tumors were of high grade and located mainly in the extremities (*n* = 18). The vast majority of patients at the time of brain metastasis diagnosis already had extracranial metastatic disease (*n* = 26). The median time from sarcoma diagnosis to cerebral metastasis diagnosis was 16 months (range, 1–136). Treatment modalities for brain metastatic disease included whole-brain radiation therapy (WBRT) (*n* = 22), chemotherapy (*n* = 17), exclusive palliative care (*n* = 5), surgery (*n* = 9), targeted therapy (*n* = 6) or stereotactic radiosurgery (*n* = 2). Most patients experienced a progression of brain metastases (*n* = 11). The median overall survival from brain metastasis diagnosis was 3 months (range, 0–80). OS was significantly influenced by time-to-brain metastases (*p* = 0.041), WBRT (*p* = 0.018), surgery (*p* = 0.002) and chemotherapy (*p* = 0.006). In a multivariate analysis, only the localization of the primary (*p* = 0.047) and WBRT (*p* = 0.038) were associated with survival with statistical significance. Patients with sarcoma brain metastases have a particularly poor prognosis and an appropriate therapeutic approach is yet to be defined.

## 1. Introduction

The brain is a rare metastatic site in patients with sarcoma and the available information is relatively limited. A large retrospective series from Memorial Sloan Kettering Cancer Center (MSKCC), including 3829 soft tissue sarcoma (STS) patients over a 17-year period, reported only 40 cases with brain metastases [1], corresponding to an incidence <1%. A more recent analysis of 5933 high-grade bone and STS patients, based on the Surveillance, Epidemiology, and End Results (SEER) database, identified brain metastases in 0.7% of patients [2]. Patients with metastatic sarcomas and those relapsing after a prolonged disease control with treatment have a higher incidence of brain metastases [3]. Notwithstanding, improvement in sarcoma systemic therapy, radiation therapy and global management has led to an increased incidence of central nervous system (CNS) metastases over time [4].

There are scarce data on the clinical presentation, treatment modalities and outcome of sarcoma brain metastatic disease, originating until recently from relatively small series and case reports [1,4,5,6,7,8]. In the last five years, two large retrospective studies have been presented with more than 100 patients in each one, from MD Anderson Cancer Center (MDACC) and the French Sarcoma Group, which have shed more light on this rare occurrence [9,10]. There is a higher tendency of some histotypes to metastasize in the brain, such as osteosarcoma [11,12] and alveolar soft part sarcoma (ASPS) [5,7,13]. Different systemic or local therapies have been described that have not been sufficiently evaluated and the optimal treatment has yet to be defined [5,12,14,15,16,17]. The prognosis is, in general, dismal, with a rather longer survival reported in patients undergoing surgery [11,12,15]. Patrikidou et al. have developed a sarcoma specific prognostic assessment index including prognostic factors such as histological subtype, performance status and the number of brain metastases [18].

We present our experience regarding patients with sarcoma who are diagnosed with brain metastases. The aim of the study was to summarize its incidence, clinical presentation, treatment modalities and outcome, as well as to examine prognostic factors associated with post-brain metastasis survival. Our results indicate that brain metastases can arise from different sarcoma histotypes and their prognosis is poor, depending mainly on the localization of the primary tumor and the therapeutic measures employed.

## 2. Patients and Methods

### 2.1. Study Design

We performed a retrospective cohort study in six cancer centers within the Hellenic Group of Sarcoma and Rare Cancers (HGSRC). Data were retrieved from the medical records of STS and bone sarcoma patients who were diagnosed de novo or developed brain metastases. The study was approved by the Scientific Committee of the HGSRC, whereas the institutional review board approval was waived due to its retrospective analysis of standard routine management. The study was conducted in accordance with the principles of the Declaration of Helsinki [19].

### 2.2. Patients Selection and Data Collection

We included patients with STS or bone sarcoma who were diagnosed de novo with brain metastasis or developed brain metastasis and were managed in the six centers between April 2010 and April 2020. All patients were ≥18 years old at brain metastasis diagnosis and the initial sarcoma diagnosis was confirmed by a tissue pathological examination.

We analyzed the histopathological, clinical and radiological data, as well as the treatment modalities and survival outcomes. Variables of interest were: histological diagnosis and grade, size of the primary tumor, age at diagnosis of the primary sarcoma, first metastasis and brain metastases, localization of the primary tumor and brain metastases, number of brain metastases, presenting symptom, treatments for brain metastases (systemic therapy, surgery and radiation therapy) and survival. Responses to treatment were defined according to Response Evaluation Criteria in Solid Tumors (RECIST) criteria [20].

Patients’ cases were discussed in the multidisciplinary tumor board and treated according to the standards of care at the time of their management, based on the following parameters: number of brain metastases, neurological symptoms, performance status (PS) of the patient and presence of extracranial disease. In summary, patients with a limited number of resectable brain metastases, good PS and absence of evolutive extracranial disease were considered to be suitable for surgical treatment. In cases of patients with non-resectable brain metastases or were not suitable for surgery, stereotactic radiotherapy (SRS) was performed. Whole-brain radiation therapy (WBRT) was administered in cases of multiple brain metastases, not accessible via surgery or not suitable for STS. Radiotherapy was also considered in cases of incomplete surgical resection.

### 2.3. Statistical Analysis

Continuous variables were summarized using descriptive statistics, including median and range values. Categorical variables were summarized using descriptive statistics, including counts and percentages.

The Kaplan–Meier method was used for survival analysis and the log-rank test was used to compare survival curves for the different types of combination therapy. Overall survival (OS) was defined as the time between the date of brain metastases diagnosis and death of any cause. Patients who were still alive were censored at the last follow-up date. Progression-free survival (PFS) was defined as the time between the date of brain metastases diagnosis and the date of progression or death of any cause, whichever came first. Patients who were still alive without disease progression were censored at the date of the last follow-up. Univariate and multivariate predictors of survival were assessed using the Cox proportional hazards model and a backward selection, by calculating hazard ratios (HR) with 95% confidence intervals (CI). We examined the following variables: gender, age at brain metastasis diagnosis, localization of the primary tumor, histotype, tumor grade, presence of extracranial disease, number of brain metastases, time-to-brain metastases, WBRT, surgery, SRS, chemotherapy and targeted therapy. Statistical analyses were computed using SPSS 26 (IBM Corp., Armonk, NY, USA). Significance was defined at *p* < 0.05.

The median follow-up of the patients after diagnosis of brain metastases was 3 months (range, 0–80) and the median follow-up after diagnosis of primary sarcoma was 22 months (range, 6–180).

## 3. Results

### 3.1. Clinicopathological Characteristics

Thirty-four adult patients with sarcoma brain metastases were included, with a male to female ratio of 16:18. The median age at diagnosis of the primary tumor was 53 years (range, 15–76) and the median age at brain metastases diagnosis was 55.5 years (range, 18–75). Table 1 summarizes the clinicopathological characteristics of the primary tumors. Twenty seven cases of STS and 7 cases of bone sarcomas were included. The most common histotypes were leiomyosarcoma (*n* = 8, 23.5%, of which one was retroperitoneal and four were uterine), Ewing sarcoma/PNET (*n* = 7, 20.6%) and osteosarcoma (*n* = 3, 8.8%). The primary tumor was located in the extremities (*n* = 18, 52.9%) and in the uterus (*n* = 5, 14.7%), presenting with a median size of 8 cm (range, 2.7–25) and with grade 3 in 25 cases (73.5%).

Nine patients (26.5%) were non-metastatic at presentation and the remaining 25 (73.5%) were stage IV. The vast majority of patients (*n* = 33, 97.1%) already had extracranial metastatic disease at brain metastases diagnosis, including 28 (82.4%) with lung metastases, 19 (55.9%) with bone metastases and 7 (20.6%) with liver metastases. Metastases were also present in other less common localizations (*n* = 12, 35.3%). Only one patient presented with de novo brain metastatic disease, concomitantly to lung metastases. Another three patients were diagnosed with brain metastases at the same time with other metastases diagnoses and the remaining 30 patients presented with brain metastases over a period of time following extracranial metastases. The median time to the first metastases was 10 months (range, 0–51) and the median time-to-brain metastases was 15.7 months (range, 0.7–136.4). The patients had received a median number of two prior systemic therapies before brain metastases diagnosis (range, 0–6). The presenting symptom was a headache (*n* = 18, 52.9%), followed by nausea (*n* = 6, 16.2%), cranial nerve disorder (*n* = 4, 11.8%), vertigo (*n* = 4, 11.8%), seizure (*n* = 2, 5.9%), hemiplegia (*n* = 1, 2.9%) or other (*n* = 2, 5.9%). It should be noted that some patients presented multiple symptoms. The Eastern Cooperative Oncology Group (ECOG) scale of Performance Status (PS) at brain metastasis diagnosis was 0 (*n* = 3), 1 (*n* = 9), 2 (*n* = 12), 3 (*n* = 6) and 4 (*n* = 4). Cerebral metastases were classified as supratentorial in the majority of patients (*n* = 20, 58.8%). A single brain metastasis was found in 13 cases (38.2%), 2–4 metastatic lesions in 11 cases (32.4%) and more than 4 in 7 cases (20.6%). Table 2 summarizes the characteristics of CNS metastases.

### 3.2. Treatment Modalities for Brain m3.2. Treatment Modalities for Brain Metastases and Outcome

Treatment modalities after brain metastases diagnosis included WBRT (*n* = 22), chemotherapy (*n* = 17), palliative care (*n* = 12), surgery (*n* = 9), targeted therapy (*n* = 6) or stereotactic radiosurgery (*n* = 2). In most patients, more than one therapeutic measure was employed (Table 3). Among the 17 patients who received chemotherapy, trabectedin was given to four patients, anthracycline-based regimen to three patients, ifosfamide/etoposide combination to four patients, vincristine/doxorubicin/cyclophosphamide alternating with ifosfamide/etoposide to four patients and paclitaxel to one patient, whereas the chemotherapeutic regimen was not available in the remaining four patients. The regimen was decided according to the sarcoma histotype. Among the six patients who were treated with targeted therapy, five received pazopanib, one received cabozantinib, whereas one of the patients with ASPS received multiple therapies.

All patients who underwent surgery for brain metastases had extracranial disease; the metastatic site was the lung in six patients, the liver in two patients, and the bone in five patients, whereas metastases were present in multiple sites in two patients. Five out of nine patients who were operated had a solitary brain metastasis, three patients had 2–4 brain lesions and the information was not available in one patient. Only two patients were managed solely with surgery (one patient with alveolar rhabdomyosarcoma and one patient with undifferentiated sarcoma). Resection of the brain metastases was followed by WBRT in six patients, SRS in two patients, chemotherapy in seven and targeted therapy in four patients.

**Figure 1:**
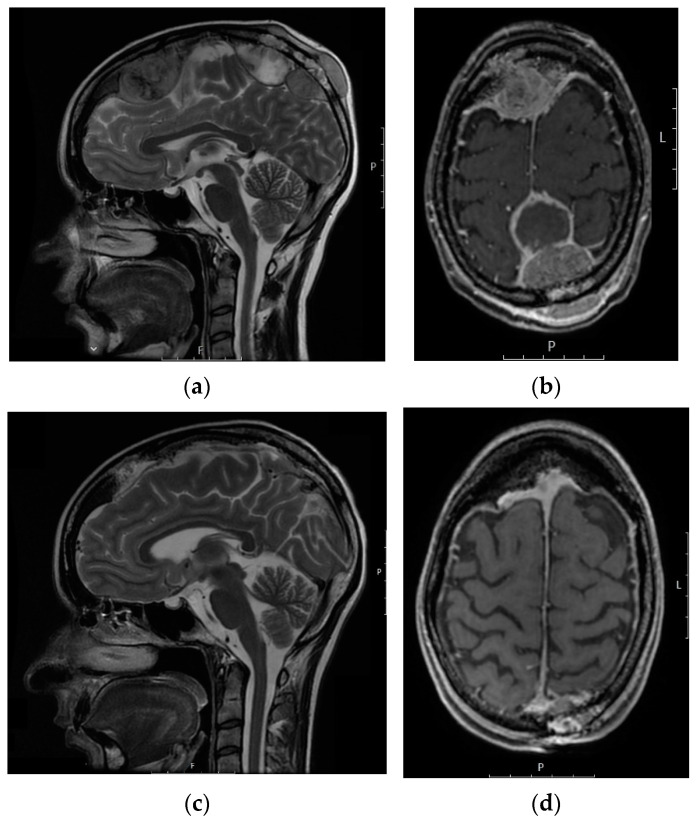
Axial T1-post IV contrast (**a**) and sagittal T2-weighted (**b**) MRI images of a 21-year old female with alveolar soft part sarcoma at the diagnosis of two large brain metastases with meningeal involvement and known skull bone metastases. The patient was treated with whole-brain radiotherapy (WBRT) and several different systemic therapies. Axial T1 (**c**) and sagittal T2-weighted (**d**) MRI images performed 2 years after WBRT, showing a very good response to treatments.

Despite these treatments, most patients experienced progression of brain metastases. In regard to CNS disease, complete response (CR) was noted in one patient, partial response (PR) in two patients, stable disease (SD) in seven patients and progressive disease (PD) in 11 patients. In the remaining 13 patients, the outcome was not evaluable because they were: (i) dead because of toxicity complications (*n* = 3), (ii) lost to follow-up (*n* = 4), (iii) dead because of extracranial PD (*n* = 3), (iv) dead because of a non-related cause (*n* = 1) or (v) dead with no further information (*n* = 2). One patient with two brain metastases from an undifferentiated sarcoma achieved a CR in CNS by surgery. The two partial responders in CNS underwent both surgery and WBRT, as well as systemic therapy (chemotherapy and targeted therapy), therefore we cannot draw any unequivocal conclusion on the efficacy of each treatment modality separately. The histotypes of these patients were leiomyosarcoma and ASPS (Figure 1). Among the seven patients with SD in CNS, three underwent WBRT and chemotherapy (doxorubicin, ifosfamide/etoposide and paclitaxel for one leiomyosarcoma, one Ewing sarcoma and one glomus tumor, respectively), one underwent surgery, one received WBRT, one received SRS and targeted therapy (pazopanib) and another one received exclusive palliative care. Among the nine operated patients, CR was noted in one patient, PR in two, SD in one and PD in four patients, while the outcome was unknown in one patient.

### 3.3. Survival and Prognostic Factors

At the time of the analysis, only three patients were still alive (one disease-free, one with intracranial PD and one with extracranial PD). Twenty patients died of their sarcoma, three patients died from treatment complications, one patient died from another reason and six patients were lost to follow-up. The toxic complications that led to death included neutropenic fever following chemotherapy, liver failure following targeted therapy in the context of advanced liver disease and cerebral hemorrhage after WBRT.

**Figure 2:**
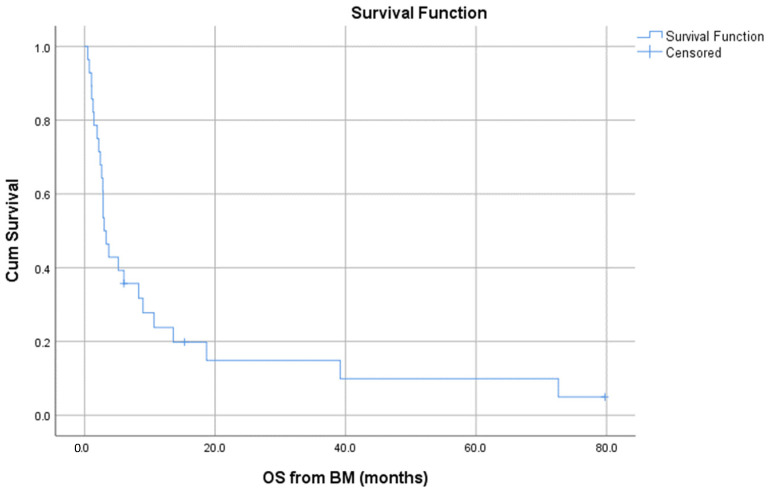
Overall survival from brain metastases diagnosis.

The median overall survival from brain metastasis diagnosis was 3 months (range, 0–80), as depicted in Figure 2. Six patients survived more than 1 year from brain metastasis diagnosis and three patients more than 3 years (long survivors).

The long survivors included two grade 2 leiomyosarcomas and one ASPS. The time-to-brain metastasis was particularly long: 42.9, 72.3 and 136.4 months. The number of brain metastases was one in two cases and two in one case. All of them had extracranial metastases as well and underwent surgery, post-operative WBRT and chemotherapy (trabectedin in two cases, regimen not available in the other one). In addition, two long survivors received targeted therapy as further line treatment.

**Figure 3:**
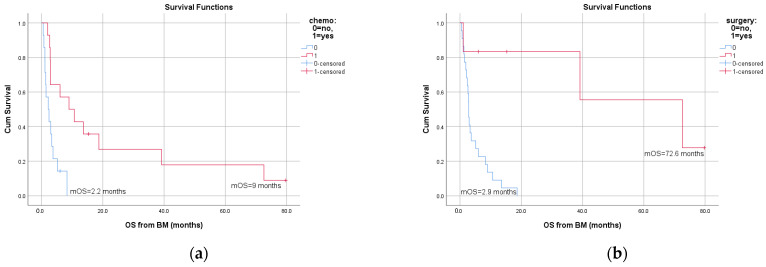

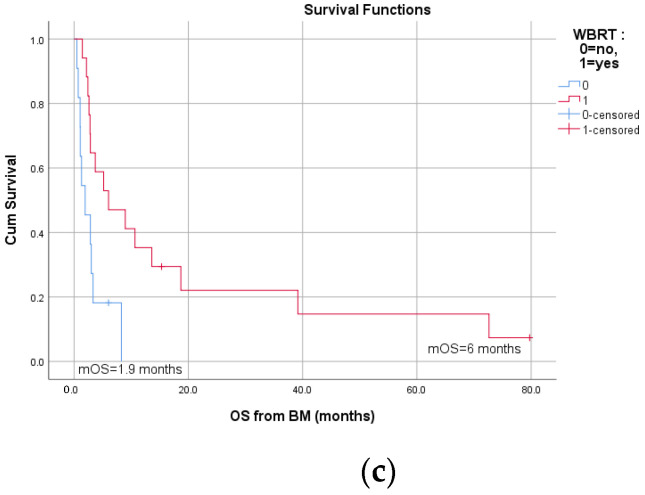
Overall survival (OS) from brain metastases according to (**a**) chemotherapy, (**b**) surgery and (**c**) whole-brain radiation therapy (WBRT).

**Figure 4:**
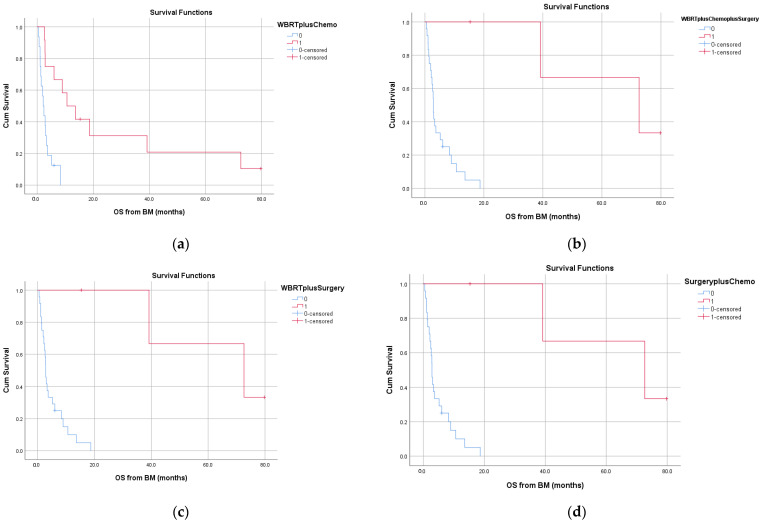
Overall survival (OS) from brain metastases according to combination therapy with (**a**) whole-brain radiation therapy (WBRT) + chemotherapy, (**b**) WBRT + chemotherapy + surgery, (**c**) WBRT + surgery and (**d**) surgery + chemotherapy.

On univariate analysis, OS was significantly influenced by time-to-brain metastases (*p* = 0.041), WBRT (*p* = 0.018), surgery (*p* = 0.002) and chemotherapy (*p* = 0.006). The significant differences in OS based on therapeutic measures are shown in Figure 3. The above parameters were also examined on multivariate analysis; only localization of the primary (*p* = 0.047) and WBRT (*p* = 0.038) were associated with survival. In addition, we assessed the effect of the different treatment combinations (WBRT + chemotherapy, WBRT + surgery + chemotherapy, WBRT + surgery and chemotherapy + surgery) on OS. Figure 4 shows the corresponding survival curves; all differences in OS were statistically significant.

## 4. Discussion

Brain imaging is not routinely included in the work-up of sarcoma patients, but only upon neurological symptoms or in certain histotypes. In the large French series, leiomyosarcoma was the most frequent subtype (18.7%), followed by Ewing sarcoma/PNET, liposarcoma, ASPS, osteosarcoma, rhabdomyosarcoma and angiosarcoma [10]. Undifferentiated sarcoma, ASPS, Ewing sarcoma and leiomyosarcoma were pointed out as the most common histotypes in the other large retrospective series from MD Anderson [9]. According to the SEER analysis, the most common subtypes were unspecified sarcoma, leiomyosarcoma, angiosarcoma, spindle cell sarcoma and osteosarcoma, in order of decreasing frequency [2]. A similar histological composition was found by two single-institution studies in Italy and Germany [12,13]. Pediatric series comprise similar histotypes, with a higher frequency of rhabdomyosarcoma [21]. ASPS has been reported to have a predisposition towards brain metastases with estimates of approximately 25% at presentation, typically occurring in young patients [14,22,23]. Osteosarcoma has also been reported to metastasize to the brain [11,17,24,25,26], presenting usually with intralesional calcifications [27]. In our study, the most common histotypes were in agreement with the previous results. Furthermore, we also found some cases of rare sarcoma subtypes, including glomus tumor, endometrial stromal sarcoma and phyllodes tumor, for which there are case reports of brain metastases [28,29,30].

Most primary tumors in our study were of a high grade, with a median diameter of 8 cm, highlighting the higher prevalence of brain metastases from sarcomas with high-risk features in general. This observation has also been pointed out by other analyses. In the MSKCC series, 39 out of 40 primary sarcomas were of a high grade, 38 were deep located and a large proportion of them (*n* = 14) had a diameter of >10 cm [1]. The majority of primary tumors in the French study also presented as grade 3 with a median tumor size of 9 cm, whereas tumor grade was significantly associated with prognosis [10]. The lack of a statistically significant prognostic association of tumor grade in our study is likely due to the small number of patients. The prognostic value of tumor grade in sarcomas, with regard to metastatic potential and mortality, has already been established [31].

The median age at diagnosis of the primary tumor and brain metastasis was 53 and 55.5 years, respectively, in accordance with the SEER analysis [2]. Patients were diagnosed at a slightly younger age in the French retrospective series [10]. In two series from MD Anderson, the median age at brain metastases diagnosis was 34.8 and 41 years, due to the inclusion of children [9,16]. A significantly younger brain metastases diagnosis age (23 years) was reported in a study of ASPS cases exclusively [7], as expected for this histotype affecting younger adults. We found a median time-to-brain metastasis of approximately 16 months, in line with previous findings. In the other series, this interval varies between 14 months and 2.2 years [4,9,10].

We did not find any remarkable predilection to any one of the sexes, with a sex ratio close to one. There is a number of studies that reported a slight male preponderance [1,2,5,9,10,12,13,16]. The low number of cases in most series does not allow drawing conclusions. In the French study, which is the largest study to date, males represented 56.5% of the population, similarly to the study from MD Anderson.

Brain metastases were classified as supratentorial in the majority of patients in our study, similarly to metastases from other cancers [32]. In the large study from MD Anderson, most cases presented with solitary parenchymal lesions, 40% of which were frontal [9]. In another single-institution neurosurgical series, all patients (*n* = 35) had supratentorial lesions [12]. Meningeal metastases appear far less frequently, constituting 3–23% of sarcoma brain metastases in the different series [1,10].

Thirty-eight percent of our patients had a solitary CNS metastasis. The number of cerebral metastases were recently found to be predictive for overall survival, in an attempt to develop a prognostic index dedicated to sarcoma brain metastases [18]; our analysis, though, did not reveal any statistically significant association of the number of metastatic foci with OS.

Synchronous metastases outside the CNS (lung, bone, liver) were noted in the vast majority of our patients (*n* = 33, 97.1%). This is a constant finding in almost all studies [2,4,5,6,7,9,10,12,16], suggesting that brain metastasis is a rather late event in the natural history of sarcoma. Gercovich et al. also noticed the occurrence of sarcoma brain metastases after a long period of disease control with first-line chemotherapy [3]. In most studies, lung metastases were already present in a large proportion of patients [1,2,4,5,7,9,10,12,15]. In the study based on the SEER database, 59% of patients with brain metastases also had lung metastases [2]. In a small study of ASPS patients, all of them developed brain metastases after lung metastases [7]. However, brain metastases can be detected at presentation of some sarcoma patients. In the MSKCC series, brain metastases were presented synchronously with the sarcoma diagnosis in 21 out of 40 patients [1] without any information regarding whether brain imaging was performed, even in asymptomatic patients. Niazi et al. reported a case of hemorrhagic cerebellar metastasis as the initial manifestation of an osteosarcoma of the lower extremity [33].

We were not able to analyze the influence of brain metastases on the prognosis—when there was an absence of extracranial disease—because of the high prevalence of patients with extracranial metastases. In an MD Anderson neurosurgical series, concurrent lung metastases had no effect on survival [15], whereas in another study, long-term survival was only observed in three out of six patients with isolated cerebral metastases versus no patients with additional metastatic sites [13]. Resection of brain metastases should not be precluded by the existence of lung metastases, as it has been demonstrated for osteosarcoma [11]. Almost half of operated patients for sarcoma brain metastases in MSKCC also underwent lung metastasectomy without any effect on survival [6].

The efficacy of each treatment modality cannot be properly evaluated because most of the patients received more than one therapy for brain metastases and because of the limited number of patients. In addition, systemic therapy for sarcomas has evolved during the 20-year study period. In total, 10 out of our 21 evaluable patients gained a clinical benefit of the different treatments, if we consider patients who experienced CR (*n* = 1), PR (*n* = 2) and SD (*n* = 7). It should be noted that the two partial responders in CNS received multimodal therapy, consisting of surgery plus WBRT, followed by chemotherapy (trabectedin for the LMS patient and the drug not available for the ASPS patient) and targeted therapy (pazopanib for the LMS patient and four different agents for the ASPS patient). In the large French study, BM were controlled in 46% of patients (12.6% PR and 7.3% CR), whereas 27% of patients who were treated with chemotherapy experienced disease control in the CNS [10]. A resolution of sarcoma brain metastases with chemotherapy has occasionally been described [34]. Furthermore, an improvement on the majority of patients’ performance status (PS) by surgery was demonstrated in a neurosurgical series of 62 patients [16].

Post-brain metastasis survival was poor in our series, with a median OS of approximately 3 months, in accordance with previous findings in which the median OS varies between 1.7 and 8 months [1,2,4,5,9,10,13]. Neurosurgical series generally report prolonged survival approaching 1 year [12,15]. These results should be interpreted with caution given the selection bias, which is rather significant. Aggressive treatment with surgery was offered to patients with a good performance status; the treatment decision was also based on disease severity.

Although WBRT, surgery, chemotherapy and time-to-brain metastases were identified as prognostic factors for OS on univariate analysis, only WBRT was confirmed on multivariate analysis. This is probably due to the small number of patients. Patrikidou et al. reported an association of PS, number of brain metastases and histology with survival [18]. Again, the modest sample size in general and per histotype and the heterogeneity of sarcomas included in our cohort probably precluded us from showing a statistically significant prognostic association of these factors. ASPS histology has been constantly associated with a good prognosis, irrespective of treatment modality [5,12,16]. In line with this observation, one of the two ASPS patients in our cohort survived for more than 3 years and received all different therapies. Some studies have shown a survival benefit from specific treatments against brain metastases, such as surgery, chemotherapy and radiation therapy [5,10]. In the study of MSKCC, the median OS in patients who underwent surgical removal of their brain metastases was 9.6 versus 2.7 months in non-operated patients [1]. However, a significantly prolonged survival was found in several analyses with treatment combinations (surgery combined with chemotherapy or radiation therapy) [4,9,17]. We also found prolonged survival with treatment combinations, including surgery plus radiation therapy and/or systemic therapy, as was the case for our long survivors.

## 5. Conclusions

We report a cohort of adult sarcoma patients with brain metastases conducted from HGSRC. Sarcomatous brain metastases are a rare event, which has not been well investigated, with limited information being available regarding factors predictive of their development. Most data originate from retrospective series and limited population-based analyses. The relatively small number of patients in these studies, including our series, should be assessed in the context of the rarity of the disease. Although brain metastases are more frequent in high-grade tumors located in the extremities and in certain histologies, it can arise from almost all sarcoma histotypes and vigilance is warranted upon neurological symptoms. In most cases, when sarcomas manifest in the CNS, lung or other metastases are already present. In contrast to brain metastases from other primary tumors, there is very limited evidence to guide treatment decisions. We showed that WBRT confers a survival benefit to patients. Previous studies have reported that local treatments of brain metastases, including surgery and radiation therapy, prolong survival. Typically, patients with sarcoma brain metastases have a particularly poor prognosis and the appropriate therapeutic approach is yet to be defined. We identified some long survivors who underwent multimodal therapy. Further research is required to evaluate the role of each therapeutic measure, as well as their sequence. Given that large prospective trials are unlikely to be performed, collaborative efforts are needed.

## Figures and Tables

**Table 1:** Characteristics of the primary sarcomas.

Characteristics	No. (%)
*Sarcoma origin*	
Soft tissue	27 (79.4)
Bone	7 (20.6)
*Localization of the primary*	
Extremities	18 (52.9)
Trunk	3 (8.8)
Retroperitoneum	1 (2.9)
Uterus	5 (14.7)
Other	7 (20.6)
*Histological type*	
Ewing/PNET	7 (20.6)
Leiomyosarcoma-soft tissue	4 (11.8)
Leiomyosarcoma-uterine	4 (11.8)
Osteosarcoma	3 (8.8)
Alveolar soft part sarcoma (ASPS)	2 (5.9)
Undifferentiated pleomorphic sarcoma (UPS)	3 (8.8)
Rhabdomyosarcoma	2 (5.9)
Synovial sarcoma	2 (5.9)
Malignant peripheral nerve sheath tumor	2 (5.9)
Other ^1^	5 (14.7)
*Grade*	
3	25 (73.5)
2	7 (20.6)
Unknown	2 (5.9)
*Stage at initial presentation*	
Localized	9 (26.5)
Metastatic	25 (73.5)

^1^ Liposarcoma, chondrosarcoma, endometrial stromal sarcoma, phyllodes tumor, glomus tumor.

**Table 2:** Characteristics of brain metastases.

Characteristic	No. (%)
*Brain metastases localization*	
Supratentorial	20 (58.8)
Infratentorial	5 (14.7)
Both supratentorial and infratentorial	5 (14.7)
Meningeal	1 (2.9)
Unknown	3 (8.8)
*Number of brain metastases*	
1	13 (38.2)
2–4	11 (32.4)
>4	7 (20.6)
Unknown	3 (8.8)

**Table 3:** Treatment modalities for patients with brain metastases.

Therapy	No. (%)
Surgery alone	2 (5.9)
Systemic therapy (chemotherapy/targeted therapy) alone	4 (11.8)
Chemotherapy (CTx)	2 (5.9)
Targeted therapy	2 (5.9)
Whole-brain radiation therapy (WBRT) alone	8 (23.6)
Surgery + WBRT	0
Surgery + systemic therapy	1 (2.9)
Surgery + systemic therapy + WBRT	6 (17.6)
WBRT + systemic therapy	8 (23.6)
Stereotactic radiosurgery (+surgery, WBRT, CTx) Exclusive palliative therapy	2 (5.9) 5 (14.7)

## Data Availability

The data presented in this study are available upon reasonable request from the corresponding author.
